# Development and Validation of Ribosomal RNA-Targeted Reverse Transcription Real-Time PCR Assays for the Sensitive and Rapid Diagnostics of High Consequence Pathogens

**DOI:** 10.3389/fmicb.2021.738868

**Published:** 2021-10-29

**Authors:** Veronika Merold, Kim Silberreis, Kilian Stoecker

**Affiliations:** Bundeswehr Institute of Microbiology, Munich, Germany

**Keywords:** diagnostics, *Yersinia pestis*, *Francisella tularensis*, real-time PCR, 16S rRNA

## Abstract

Real-time PCR (rtPCR) has become an essential tool in clinical microbiology and has been used for the acute diagnostics of many pathogens. Key performance indicators of rtPCR assays are their specificity as well as their analytical and clinical sensitivity. One way to maximize the sensitivity of such diagnostic rtPCRs is the use of genomic targets, which are present in several copies in the target cells. Here, we use the naturally pre-amplified ribosomal RNA as target for specific and highly sensitive reverse-transcription rtPCR detection of two high consequence pathogens, *Yersinia pestis* and *Francisella tularensis*. We determined their analytical sensitivity and illustrate that the newly designed assays are superior compared with other previous published rtPCR assays. Furthermore, we used spiked clinical sample matrices to evaluate their clinical applicability. Finally, we demonstrate that these assays can be applied on heat-inactivated samples without the need of time-consuming nucleic acid extraction.

## Introduction

Rapid, accurate, and sensitive detection of pathogenic bacteria is crucial for diagnostics of infectious diseases. This is especially the case for high consequence pathogens such as the biological agents *Francisella tularensis* and *Yersinia pestis*. For neither of these notorious pathogens a vaccination exists and they cause severe diseases, which require swift application of medical countermeasures (Boisset et al., 2014; Pechous et al., 2016). Traditional approaches for detection and identification of pathogenic bacteria include microbial cultivation methods, serological assays, and nucleic acid detection–especially via polymerase chain reaction (PCR). Although culture is very sensitive, and still often regarded as the gold standard for diagnostics of certain bacterial pathogens, it is very time consuming and involves extensive biosafety measures. Moreover, bacterial culture is often not successful–for example, *F. tularensis* is a fastidious and slow-growing organism and thus can be cultured only from less than 10% of tularemia patients (Maurin and Gyuranecz, 2016). For *Y. pestis*, cultivation is often aggravated by administration of antibiotics prior to sampling (Vallès et al., 2020). With regard to serology, detection of patient antibodies is only possible days to weeks after symptom onset, due to the time needed for seroconversion (Maurin and Gyuranecz, 2016; Vallès et al., 2020). Another option for direct pathogen detection are immunochromatographic rapid tests, such as lateral flow assays. However, such assays often lack the needed sensitivity (Ziegler et al., 2021) and are thus not suitable for reliable diagnostics. In contrast, real-time PCR (rtPCR) represents a rapid, highly sensitive, and specific method. In the last decade, rtPCR has become an essential tool in the laboratory diagnostics of human pathogens (Maurin and Gyuranecz, 2016). The analytical sensitivity–often expressed as limit of detection (LOD)–is a key factor of paramount importance for every diagnostic rtPCR assay. The theoretical LOD of PCR is three targets per reaction (Wittwer and Kusukawa, 2004; Espy et al., 2006). However, many factors such as nucleic acid extraction and amplification efficiency and/or presence of PCR inhibitors negatively affect the analytical sensitivity, resulting in reduced clinical sensitivity (i.e., the ability of an assay with a given analytical sensitivity to detect a pathogen in a clinical sample and thus correctly diagnose the respective disease). The use of multi-copy targets can compensate for these negative factors (Klee et al., 2006). Such a multi-copy target is ribosomal RNA (rRNA), which is naturally pre-amplified in bacterial cells where it makes up to 80% of the cell’s total RNA. Moreover, it is less prone to degradation than messenger RNA. Depending on the target bacterium, there are between 100 and 10,000 ribosomes per cell (Woese, 1987). Several studies have already shown that reverse transcription rtPCR (RT-rtPCR) targeting rRNA molecules enables the detection of bacteria with 100 times the sensitivity of DNA-targeted rtPCR because of the high copy number of targeted rRNA molecules (Cox et al., 2003; Matsuda et al., 2007; Kubota et al., 2010; Choi et al., 2015). However, due to the lack of specific sequence variations, rRNA genes and their transcripts are often deemed unsuitable for the unambiguous identification of many pathogens (Chase et al., 2005). Accordingly, the only hitherto existing rtPCR targeting the 16S rRNA gene of *Y. pestis* failed to discriminate between *Y. pestis* and other bacteria of the genus *Yersinia* (Tomaso et al., 2003). For *F. tularensis*, a rtPCR successfully targeting the 16S rDNA has been described (Knüpfer et al., 2020). However, in its present form, the sensitivity of this assay is about 86 colony forming units (CFU)/ml (Knüpfer et al., 2020) and thus not optimal.

In this study, we established and validated highly sensitive RT-rtPCRs for high consequence pathogens targeting single nucleotide polymorphisms in their 16S/23S rRNA. Furthermore, these assays were also validated for clinical sample matrices. Besides extracted nucleic acids as template, heat-inactivated samples were added directly to the reaction mix. By omitting the time-consuming nucleic acid extraction process, it was shown that with the presented assays rapid diagnostics of *Y. pestis* and *F. tularensis* with unmatched sensitivity are possible.

## Methods

### Reference Strains and Culture Conditions

The used strains of *Y. pestis* EV76, *Yersinia pseudotuberculosis* YPII, and *F. tularensis* F049 are part of the bacterial strain collection of the Bundeswehr Institute of Microbiology. *F. tularensis* F049 was cultured aerobically in liquid Mueller–Hinton II broth (Becton & Dickinson, Heidelberg, Germany) supplemented with 2% IsoVitaleX (Becton & Dickinson) at 37°C for 48 h by 125 rpm or on Mueller–Hinton II agar (Becton & Dickinson) for 72 h at 37°C. *Y. pestis* EV76 was cultured aerobically in liquid LB broth (Becton & Dickinson) at 27°C for 24 h by 125 rpm or on LB agar for 72 h at 37°C. *Y. pseudotuberculosis* YPII was cultured in liquid LB broth at 37°C for 24 h by 125 rpm. All cultivation was conducted under BSL 2 conditions.

### Determination of Bacterial Cell Numbers by Plate Count Method and Direct Microscopy-Based Counting

For the quantification of bacteria cells in pure culture, inoculums of *Y. pestis* and *F. tularensis* were incubated at corresponding culture conditions for 20 h. After that, cultures were adjusted to McFarland 0.5 in 0.9% NaCl (Merck, Darmstadt, Germany), respectively, to OD_600_ of 0.1. A serial 10-fold dilution was prepared in corresponding growth media and 100 μl of each dilution was plated in triplicates on the corresponding agar for 72 h. The colonies on the agar plates were counted and the concentration of the initial culture was calculated. In addition, the serial 10-fold dilution of the bacteria cultures was quantified microscopically by phase-contrast microscopy.

### Development of Ribosomal RNA Gene-Targeted Primers

All primers and probes are listed in Table 1. The *Y. pestis* specific probe has been modified from the study of Aistleitner et al. (2020). To avoid unspecific results in closely related bacteria like *Y. pseudotuberculosis*, a competitive probe was designed (YP23S_CP). *F. tularensis* specific probe Ftul_TM from Knüpfer et al. (2020) was redesigned reverse complementary. The corresponding primer sets were designed using Geneious software (version 2020). For each target, 10 pairs of different primers were initially designed. Primers were checked for primer dimer formation by running a SYBR green–based rtPCR with subsequent melting curve analyses (data not shown) and the best-performing primer pair was finally selected. The position of the finally selected target sites for *F. tularensis* is nucleotides 1,086–1,197 on the *F. tularensis* 16S rRNA gene sequence (GenBank accession no. NR_029362.1) and for *Y. pestis* 1,503–1,619 on the *Y. pestis* KIM 10 + 23S rRNA gene sequence (GenBank accession no. NR_076183). The specificity of the designed primers was confirmed by comparing the sequences with the BLAST program of the National Center for Biotechnology Information (NCBI) as well as by submitting the sequences to the probe match program of the Ribosomal Database Project (RDP-II).

**TABLE 1:** Primers and probes used in this study.

**Bacteria**	**Target**	**Sequence**	**Product size (bp)**	**Source**
*Y. pestis*	*pla*	*Pla*_F: 5′-GTA ATA GGT TAT AAC CAG CGC TT	232	Riehm et al., 2011
		*Pla*_R: 5′-AGA CTT TGG CAT TAG GTG TG		
		*Pla*_TM: 5′-6FAM-ATG CCA TAT ATT GGA CTT GCA GGC CAG T–BBQ		
*Y. pestis*	23S rRNA	YP23S_R: 5′-GCT TAT CAA CCC TGA GGC GT	117	This study
		YP23S_F: 5′-GTG TCG GTT TGG GGT ACG AT		
		YP23S_TM: 5′-6FAM-CTG CTT CTG CAC CGT G GTG–BBQ		
		YP23S_CP: 5′-AA CTG CTT CTG CAC CGT A GTG–PH		
*F. tularensis*	16S rRNA	Ftul16S_F: 5′-AAC GAG CGC AAC CCC TAT TGA T	112	This study
		Ftul16S_R: 5′-TAA GGG CCA TGA TGA CTT GAC G		
		Ftul16S_TM: 5′-Cy5-GCC TTG TCA GCG GCA GTC TTA ATA G–BBQ		
*F. tularensis*	16S rRNA	Ftul_F: 5′-GAG CGC AAC CCC TAT TGA TA	184	Knüpfer et al., 2020
		Ftul_R: 5′-TTT TTG AGT TTC GCT CCA GCT		
		Ftul_TM: 5′-6FAM-CTA TTG AGA CTG CCG CTG ACA AGG C–BBQ		

### Collection and Preparation of Clinical Samples

All clinical samples were diagnostic residual samples by the central diagnostic service of the Bundeswehr Institute of Microbiology. All clinical samples were leftover material from diagnosing of patients, which would have been discarded otherwise. No extra sampling from the patients was performed. The work with clinical samples has been carried out in-line with “The Code of Ethics of the World Medical Association (Declaration of Helsinki)” and according to good clinical practice guidelines. According to the local legislation, no formal approval of a research ethics committee was needed because there were anonymized and for research purposes stored samples used. Blood culture and pleural puncture samples were collected in BD aerobic BACTEC Plus blood culture bottles (Becton & Dickinson). They were obtained from patients with pneumonia, confirmed to be caused by pathogens other than *F. tularensis* or *Y. pestis*. The peripheral blood was sampled in EDTA–Monovettes (Sarstedt, Nümbrecht, Germany) and throat swabs were obtained with Amies media containing FLOQ Swabs (Copan, Murrieta, Canada). Sputum samples were pooled and stored at −80°C for a maximum of 2 months. Blood culture and pleural puncture samples were stored at room temperature for 1 week and EDTA–blood was processed immediately after the collection. The different samples were spiked with serial 10-fold dilution of *Y. pestis* or *F. tularensis* bacteria from 10^3^ to 0.1 cells/ml. To get rid of inhibitors in EDTA–blood, the samples were centrifuged at 500 × *g* for 3 min to generate plasma. Plasma was used for RNA and DNA extraction or was directly added to PCR after heat inactivation.

### Isolation of DNA and Real-Time Polymerase Chain Reaction

DNA extraction was performed by DNeasy Blood and Tissue Kit (Qiagen GmbH, Hilden, Germany). For this, 500 μl of bacteria culture or clinical sample (maximum 10^9^ cells/ml) was processed according to the manufacturer’s instructions. DNA was eluted in 50 μl nuclease-free water and stored at −25°C.

The rtPCR was conducted using 1 × qPCR Master Mix (Biotechrabbit, Berlin, Germany) with 0.4 μM primer F, 0.4 μM primer R, and 0.4 μM probe (TIB Molbiol, Berlin, Germany). The amplification program of *Y. pestis* and *F. tularensis* consisted of one cycle at 95°C for 2 min, followed by 40 cycles at 94°C for 8 s, 60°C for 8 s, and 72°C for 12 s. The fluorescent products were detected in the last step of each cycle. To each PCR 5 μl of DNA or pure culture was added. Amplification and detection were performed in magnetic induction cycler MIC (Bio Molecular Systems, Upper Coomera, Australia).

### Isolations of RNA and Reverse Transcription Real-Time Polymerase Chain Reaction

For RNA stabilization, two volumes of RNAprotect bacterial reagent (Qiagen) were added to the fresh culture of each bacterial strain or clinical sample (500 μl), mixed, and incubated for 5 min at room temperature. After centrifugation at 5,000 × *g* for 10 min, the supernatant was discarded, and the pellet was stored at −80°C until it was used for extraction of RNA. For this, a thawed sample was processed according to the manufacturer’s instructions by RNeasy Protect Bacteria kit (Qiagen). The following DNase treatment was skipped in this study because it was confirmed that untreated and DNase-treated samples showed identical results in the preliminary experiments, indicating that contaminating DNA does not affect RT-rtPCR quantification (data not shown). Isolated RNA is stored at −80°C until further usage.

The RT-rtPCR analysis was conducted with one-step reactions using the OneStep RT-PCR kit (Qiagen). The master mix compositions were mixed according to the manufacturer’s manual for 25 μl in total with 0.4 μM primer F, 0.4 μM primer R, and 0.4 μM probe (TIB Molbiol). For the *Y. pestis* assay 1.2 μM of competitor probe was added. The reaction mixture was incubated at 50°C for 30 min for reverse transcription. The continuous amplification program for *Y. pestis* and *F. tularensis* consisted of one cycle at 95°C for 15 min, followed by 40 cycles at 94°C for 30 s, 60°C for 30 s, and 72°C for 60 s. The fluorescent products were detected in the last step of each cycle. To each rtPCR 5 μl of RNA or pure culture was added. Amplification and detection were performed in magnetic induction cycler MIC (Bio Molecular Systems).

### Heat Inactivation of Samples

To streamline the diagnostic process, it was tested if samples can be used directly for rtPCR without nucleic acid extraction. Therefore, 500 μl of the samples (bacterial pure culture or clinical sample) was heated up to 95°C for 10 min. Inactivation was demonstrated by plating out the treated cultures as growth control.

### Validation of Real-Time Polymerase Chain Reaction and Reverse Transcription Real-Time Polymerase Chain Reaction Assays

The validation was performed according to the MIQE guidelines (Bustin et al., 2009). To validate the new rRNA-targeted PCR assays, linearity, analytical and diagnostic specificity, LOD, and repeatability and reproducibility were determined. Linearity was determined using serial 10-fold dilutions of heat-inactivated culture or nucleic acid extracted from culture at concentrations 10^6^ to 10^0^ cells/ml. At each concentration, three replicates were tested in a single run. The linear range of this plot is the linear dynamic range of the rtPCR assay. The assay performance or amplification performance was calculated from the slope of linear regression. The linearity was indicated by the coefficient of determination (*R*^2^) value. The repeatability and reproducibility were tested by intra- and inter-assays. The inter-assay was performed with the same concentration as in intra-assay at three different days and three different experiments. Sensitivity was determined as LOD95 value (Bustin et al., 2009). Therefore, five replicates of different concentrations were measured and analyzed by Probit regression model in R (Uhlig and Gowik, 2018). To determine the analytical specificity, 44 different bacteria (Supplementary Table 1) that are of differential diagnostic relevance for pulmonary infections were subjected to the *Y. pestis* 23S rRNA-gene and *F. tularensis* 16S rRNA-gene assays. In addition, further strains, six for *Y. pestis* and seven for *F. tularensis*, were evaluated via rtPCR (Supplementary Table 2 and Supplementary Figure 1). The clinical matrix background specificity was checked by subjecting three different samples in the *Y. pestis* assay (blood culture, sputum, and blood plasma) and four different samples in the *F. tularensis* assay (blood culture, blood plasma, pleural puncture, and swab sample).

### Statistical Analysis

The data preparation and statistical analysis were performed with R studio (RStudio, version 1.3.1056). Presentations were created with BioRender.^1^ Regression analysis was performed to determine the statistical correlations of the results, and Pearson’s correlation coefficient was calculated. The intra- and inter-assay variability was calculated with the coefficient of variance. Sensitivities and specificities of assays were calculated as proportions of positive and negative cases. A Probit regression analysis was performed to determine the LOD (Uhlig and Gowik, 2018).

## Results

### Primer Specificity

**FIGURE 1:**
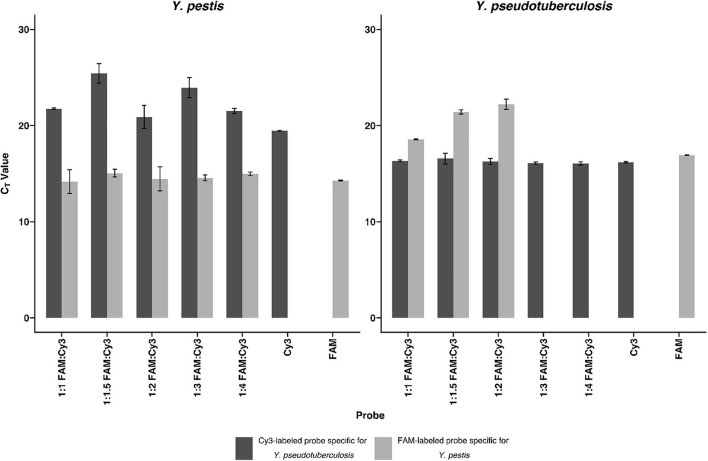
Competitive probe detecting *Yersinia pseudotuberculosis* in comparison with the probe detecting *Yersinia pestis*. To show the effectiveness of the competitive probe, both probes were tested separately and in different ratios of the probes for *Y. pestis* and *Y. pseudotuberculosis*. DNA (0.5 ng/μl) isolated from both bacteria were subjected to rtPCR. The competitive probe is labeled with Cyanine 3 (Cy3), whereas the *Y. pestis* specific probe is labeled with carboxyfluorescein (FAM). The detection of *Y. pestis* is not notably influenced by the addition of the competitive probe. *Y. pseudotuberculosis* is undetectable in the FAM channel using a ratio of 1:3 *Y. pestis* specific probe: *Y. pseudotuberculosis* specific probe or higher. Error bars represent the SD of three independent replicates.

The analytical and diagnostic specificity of the newly developed primers were evaluated by applying the assays to DNA (1 ng/μl) of 44 different bacteria of differential diagnostic relevance for pulmonary infections (Supplementary Table 1). Each primer–probe set gave positive results only for its respective bacterial target species, regardless of which target strain was analyzed (Supplementary Table 2 and Supplementary Figure 1). To ensure specific discrimination between the two closely related species *Y. pestis* and *Y. pseudotuberculosis*, a competitor probe was designed. This competitor probe has full match to the respective 23S rRNA target site of *Y. pseudotuberculosis*. When applied in a 1:3 ratio, it prevents unspecific binding and facilitates specific *Y. pestis* detection (Figure 1).

To determine the diagnostic specificity, the following samples, which previously tested negative for *Y. pestis* and/or *F. tularensis*, were analyzed (as heat-inactivated samples as well as isolated RNA and DNA): from pneumonia patients two pleural puncture samples, two blood cultures, one EDTA-blood sample, and one throat-swab sample. Furthermore, one skin sample of a tularemia suspect patient was analyzed. None of those negative clinical samples gave a (false) positive result with the newly designed assays.

### Efficiency, Dynamic Range, Precision, and Limits of Detection

**FIGURE 2:**
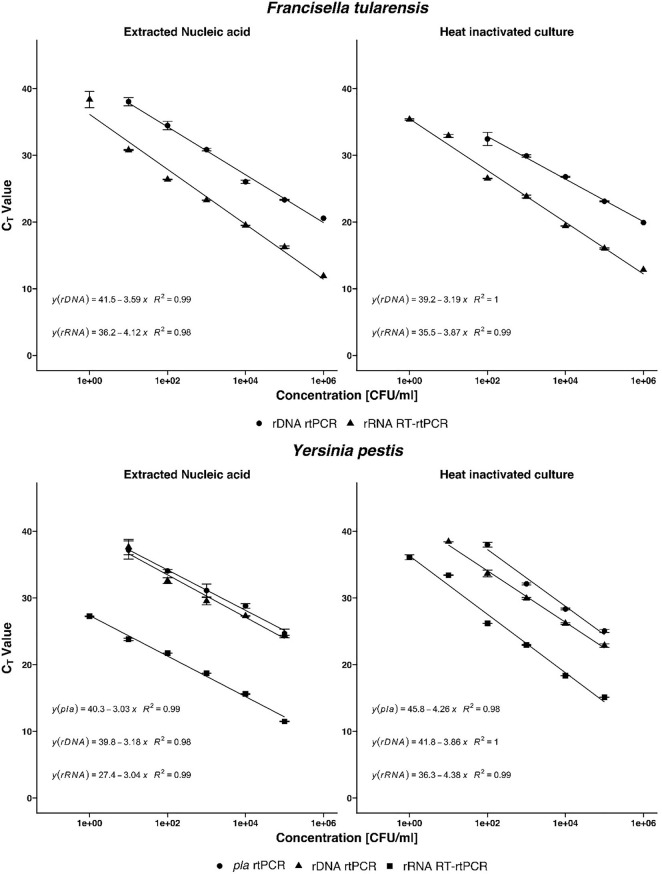
Detection of *Francisella tularensis* and *Yersinia pestis* by real-time PCR (rtPCR) and reverse transcriptase (RT)-rtPCR. In a base-10 semi-logarithmic graph, the threshold cycle is plotted versus the concentration in colony-forming units per milliliter (CFU/ml) and the data are fitted to a straight line. Coefficient of determination (*R*^2^) is determined by linear regression analysis, indicated by the respective formula. Comparison of *F. tularensis* rDNA rtPCR and rRNA RT-rtPCR as well as *Y. pestis pla* rtPCR, rDNA rtPCR, and rRNA RT-rtPCR. Error bars represent the SD of three independent replicates.

To determine the linear dynamic range of the assays, serial 10-fold dilution from 10^0^ to 10^6^ CFU/ml of heat-inactivated culture or nucleic acid extracted from culture were analyzed (Figure 2). For all assays, the coefficient of determination (*R*^2^) was >0.98, indicating a precise log-linear correlation between the input sample and the number of transcripts detected. The amplification efficiencies of RT-rtPCR and rtPCR for heat-inactivated culture samples are <90%, and the amplification efficiencies of both PCR assays analyzing extracted nucleic acids are between 90 and 114%. The precision of the different rtPCR assays was calculated from three replicates of three independent dilutions. Mean cycle threshold (C_*T*_) values and coefficient of variation (CV) were calculated from each dilution. There is a high repeatability for the different assays with CVs between 0.09 and 3.69% and high reproducibility with CVs between 0.75 and 5.91% of the calculated crossing points in the range of linearity. To determine the analytical sensitivity for the newly developed rRNA-targeted assays, dilutions of extracted nucleic acids and heat-inactivated pure culture samples were analyzed. The LODs are expressed as colony-forming units per milliliter and determined by Probit analyses (Table 2). LODs for the rRNA-targeted RT-rtPCR ranged between 1.17 CFU/ml for heat-inactivated *Y. pestis* to 0.22 CFU/ml for *F. tularensis*. For rDNA-targeted rtPCR, LODs were for *F. tularensis* below 14.99 CFU/ml and for *Y. pestis* below 13.24 CFU/ml. In general, the sensitivities of rRNA targeted RT-rtPCR assays are about 50 times higher than the sensitivities of the rDNA rtPCR assays.

**TABLE 2:** Sensitivities of rRNA- and rDNA-targeted PCR assays of *F. tularensis* and *Y. pestis* determined by Probit analysis.

**Bacteria**	**Assay**	**Extracted nucleic acid**	**Heat-inactivated culture**
*F. tularensis*	16S rRNA RT-rtPCR	0.22 CFU/ml (0.166934, 0.321908)	0.22 CFU/ml (0.166934, 0.321908)
	16S rDNA rtPCR	12.29 CFU/ml (10.5709, 238.815)	14.99 CFU/ml (8.25633, 36.0172)
*Y. pestis*	23S rRNA RT-rtPCR	0.78 CFU/ml (0.623249, 1.39149)	1.17 CFU/ml (0.648735, 0.648735)
	23S rDNA rtPCR	11.79 CFU/ml (10.3363, 23.3874)	13.24 CFU/ml (9.3294, 39.0843)

### Validation of Ribosomal RNA-Targeted Reverse Transcription Real-Time Polymerase Chain Reaction Assays on Clinically Relevant Samples

**FIGURE 3:**
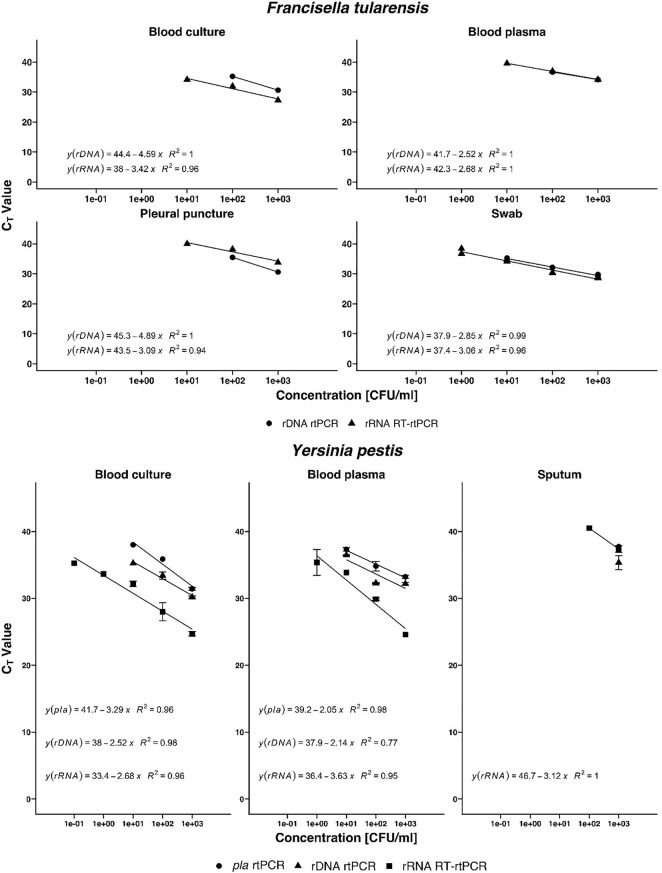
Validation of the new real-time PCR (rtPCR) and reverse transcriptase (RT)-rtPCR assays, respectively, for *Francisella tularensis* and *Yersinia pestis* with clinical sample matrices using blood culture, blood plasma, pleural puncture, swab, and sputum. Coefficient of determination (*R*^2^) is determined by linear regression analysis, indicated by the respective formula. Error bars represent the SD of three independent replicates.

For the validation with clinical sample matrices, serial dilutions of *F. tularensis* were spiked into tularemia-negative blood culture, blood plasma, pleural puncture, and throat swab samples. *Y. pestis* bacteria were spiked into plague-negative blood culture, blood, and sputum samples. The bacteria were harvested after 20 h of growth and spiked to the aforementioned sample matrices to a final concentration of 10^3^ to 10^–1^ cells/ml. To facilitate fast and easy diagnostics, the time-consuming nucleic acid extraction step was omitted and heat inactivation of the respective samples was used only. rRNA-targeted assays were usually one log step more sensitive than rDNA and, in the case of *Y. pestis*, the *pla* targeted assay (Figure 2). *F. tularensis* and *Y. pestis* in clinical sample matrices could be detected one log step more sensitive by RT-rtPCR than by rtPCR (Figure 3). *Y. pestis* was detected in blood culture, and blood and sputum samples, by rRNA RT-rtPCR, rDNA rtPCR, and *pla* rtPCR. Linear regression equation could not be determined for rDNA and *pla* assays analyzing sputum samples because only one concentration could be determined.

## Discussion

*Yersinia pestis* and *Francisella tularensis* are both dangerous infectious agents with worldwide zoonotic prevalence (Faber et al., 2018). Consequently, there is a huge variety of assays for their diagnosis. For plague diagnostics, world health organisation (WHO) still defines the bacterial culture as the gold standard (Vallès et al., 2020). However, the associated drawbacks of cultivation-based diagnostics (time consuming, mandatory biosafety measures, etc.) do not match the medical needs for rapid diagnostic tests. As the existing immunochromatographic test suffers from unspecificities (Rajerison et al., 2020), rtPCR methods have been identified as highly valuable alternatives in plague diagnostics (Vallès et al., 2020). Due to the challenging clinical sample matrices (sputum, bubo aspirate, and blood), a robust assay with high analytical sensitivity is a prerequisite. Yet existing assays seem to suffer from insufficient sensitivity (Randremanana et al., 2019) despite the fact that the target of one of these assays, the *pla* gene located on the pPCP1 plasmid, is already a multi-copy target with numbers varying between 18 (Rajanna et al., 2010) and 52.3 pPCP1 plasmids per target cell (Müller et al., 2021). Moreover, this target may be found in other *Enterobacteriaceae* such as *Citrobacter koseri* and *Escherichia coli* (Hänsch et al., 2015). Hence, results obtained from this assay always need to be confirmed by an additional rtPCR targeting, for instance, the caf1 gene (Engelthaler et al., 1999). Our newly designed assay has an analytical sensitivity of 0.78 CFU/ml (extracted nucleic acids) and 1.17 CFU/ml (heat-inactivated culture) and thus is one log more sensitive than the previous existing gold standard PCR assay. For the spiked clinical sample matrices, the LODs are slightly higher but the trend remains the same, with the rRNA targeted RT-rtPCR representing the most sensitive assay. Interestingly, the difference between the CT values between rRNA and DNA assays is less pronounced for the clinical matrix samples, when compared with the pure culture. Presumably, the reverse transcription is more prone to inhibitory aspects of the clinical samples. This, however, has no influence on the overall LOD. Sputum proved to be the most challenging sample, with LODs of 1 × 10^2^ and 1 × 10^3^ CFU/ml for rRNA and rDNA or *pla*, respectively. Again, the rRNA-targeted approach was superior to the rDNA- or *pla*-targeted assays.

For *F. tularensis*, positive cultures are rarely obtained (Hepburn and Simpson, 2008). Thus, tularemia diagnostics are usually serological (Maurin et al., 2011). However, during the first 2 weeks of the disease, there are usually no significant antibody titers, rendering serology unsuitable for acute diagnostics (Maurin et al., 2011). For the rapid detection from lymph node samples, blood culture or swab samples rtPCR proved to be a valuable technology (Maurin et al., 2010). The ability to detect *F. tularensis* rapidly and with a high sensitivity is also important in the context of protection against bioterrorist attacks (Dennis et al., 2001). Whereas previously described PCR assays have LODs ranging from 10 genome equivalents (Kugeler et al., 2006) to 86 CFU/ml (Knüpfer et al., 2020), our newly designed RT-rtPCR assay is able to detect 0.22 CFUs and is thus to our knowledge the analytically most sensitive PCR assay yet described. In its present form, the assay is unable to discriminate between the highly pathogenic *F. tularensis* subspecies (e.g., *holarctica* or tularensis A.1) and the less pathogenic *Francisella philomiragia* or *F. tularensis* subspecies *novicida*. In comparison with the original assay developed by Knüpfer et al. (2020), the newly designed probe in this study has a weak mismatch, which could be used to design a competitor probe for further discrimination. As for the *Y. pestis* assay, the LODs for the spiked clinical matrices were higher compared with pure culture experiments. Furthermore, the differences between the CT values of rRNA and rDNA targeted assays vanished nearly completely. Regardless of this observation, the rRNA-targeted assay maintained the higher overall sensitivity and detected *F. tularensis* in all tested clinical matrices with one log step more than the rDNA assays.

Reverse transcription real-time PCR has the disadvantage that it takes longer compared with conventional rtPCR due to the inevitable reverse-transcription step. This step accounts usually for 20–50 min (in our case 30 min), depending on the respective reverse transcriptase used and the protocol. To compensate this delay, the time-consuming RNA extraction step was omitted and the sample was simply heat inactivated. Obviously, this heat inactivation is enough to release sufficient rRNA from the target cells. Despite the fact that no adverse effects were observable in this study, this procedure might not be suitable for all types of clinical samples and has to be evaluated for each clinical sample type specifically. Furthermore, the advent of ultra-fast PCR technologies such as pulsed controlled amplification (Müller et al., 2021) make the delay caused by reverse-transcription negligible.

In summary, as in previous studies (Cox et al., 2003; Matsuda et al., 2007; Kubota et al., 2010; Choi et al., 2015), RT-rtPCR targeting the rRNA proved to be superior in terms of analytical and clinical sensitivity, compared with all yet published respective DNA-targeted PCR assays. This was demonstrated for two high consequence pathogens for which such highly sensitive assays were until now lacking. The successful use of a competitor probe to achieve discrimination of *Y. pestis* from the closely related *Y. pseudotuberculosis* based on a single nucleotide variation paves the way for new potential rRNA target sites.

## Data Availability Statement

The raw data supporting the conclusions of this article will be made available by the authors, without undue reservation.

## Ethics Statement

Ethical review and approval was not required for the study on human participants in accordance with the Local Legislation and Institutional Requirements. Written informed consent for participation was not required for this study in accordance with the National Legislation and the Institutional Requirements.

## Author Contributions

KSt conceptualized the study and designed the experiments. VM and KSi conducted the experiments. KSt, VM, and KSi analyzed and interpreted the results. KSt and VM wrote the first draft. KSt and KSi edited the manuscript. All authors contributed to the article and approved the submitted version.

## Conflict of Interest

The authors declare that the research was conducted in the absence of any commercial or financial relationships that could be construed as a potential conflict of interest.

## Publisher’s Note

All claims expressed in this article are solely those of the authors and do not necessarily represent those of their affiliated organizations, or those of the publisher, the editors and the reviewers. Any product that may be evaluated in this article, or claim that may be made by its manufacturer, is not guaranteed or endorsed by the publisher.

## Footnotes

1
BioRender.com

## References

[B1] Aistleitner K. Sieper T. Stürz I. Jeske R. Tritscheller S. Mantel S. (2020). NOTIFy (non-toxic lyophilized field)-FISH for the identification of biological agents by Fluorescence in situ Hybridization. *PLoS One* 15:e0230057. 10.1371/journal.pone.0230057 32142548 PMC7059943

[B2] Boisset S. Caspar Y. Sutera V. Maurin M. (2014). New therapeutic approaches for treatment of tularaemia: a review. *Front. Cell Infect. Microbiol.* 4:40. 10.3389/fcimb.2014.00040 24734221 PMC3975101

[B3] Bustin S. A. Benes V. Garson J. A. Hellemans J. Huggett J. Kubista M. (2009). The MIQE guidelines: minimum information for publication of quantitative real-time PCR experiments. *Clin. Chem.* 55 611–622. 10.1373/clinchem.2008.112797 19246619

[B4] Chase C. J. Ulrich M. P. Wasieloski L. P. Jr. Kondig J. P. Garrison J. Lindler L. E. (2005). Real-time PCR assays targeting a unique chromosomal sequence of *Yersinia pestis*. *Clin. Chem.* 51 1778–1785. 10.1373/clinchem.2005.051839 16099940

[B5] Choi Y. Hong S. R. Jeon B. Y. Wang H. Y. Lee G. S. Cho S. N. (2015). Conventional and real-time PCR targeting 16S ribosomal RNA for the detection of *Mycobacterium tuberculosis* complex. *Int. J. Tuberc. Lung Dis.* 19 1102–1108. 10.5588/ijtld.14.0472 26260833

[B6] Cox C. J. Kempsell K. E. Gaston J. S. (2003). Investigation of infectious agents associated with arthritis by reverse transcription PCR of bacterial rRNA. *Arthrit. Res. Ther.* 5 R1–R8. 10.1186/ar602 12716447 PMC154423

[B7] Dennis D. T. Inglesby T. V. Henderson D. A. Bartlett J. G. Ascher M. S. Eitzen E. (2001). Tularemia as a biological weapon: medical and public health management. *JAMA* 285 2763–2773. 10.1001/jama.285.21.2763 11386933

[B8] Engelthaler D. M. Gage K. L. Montenieri J. A. Chu M. Carter L. G. (1999). PCR detection of *Yersinia pestis* in fleas: comparison with mouse inoculation. *J. Clin. Microbiol.* 37 1980–1984. 10.1128/JCM.37.6.1980-1984.1999 10325359 PMC85002

[B9] Espy M. J. Uhl J. R. Sloan L. M. Buckwalter S. P. Jones M. F. Vetter E. A. (2006). Real-time PCR in clinical microbiology: applications for routine laboratory testing. *Clin. Microbiol. Rev.* 19 165–256. 10.1128/CMR.19.1.165-256.2006 16418529 PMC1360278

[B10] Faber M. Heuner K. Jacob D. Grunow R. (2018). Tularemia in Germany-A re-emerging zoonosis. *Front. Cell Infect. Microbiol.* 8:40. 10.3389/fcimb.2018.00040 29503812 PMC5821074

[B11] Hänsch S. Cilli E. Catalano G. Gruppioni G. Bianucci R. Stenseth N. C. (2015). The pla gene, encoding plasminogen activator, is not specific to *Yersinia pestis*. *BMC Res. Notes* 8:535. 10.1186/s13104-015-1525-x 26438258 PMC4593223

[B12] Hepburn M. J. Simpson A. J. (2008). Tularemia: current diagnosis and treatment options. *Expert Rev. Anti. Infect. Ther.* 6 231–240. 10.1586/14787210.6.2.231 18380605

[B13] Klee S. R. Tyczka J. Ellerbrok H. Franz T. Linke S. Baljer G. (2006). Highly sensitive real-time PCR for specific detection and quantification of *Coxiella burnetii*. *BMC Microbiol.* 6:2. 10.1186/1471-2180-6-2 16423303 PMC1360083

[B14] Knüpfer M. Braun P. Baumann K. Rehn A. Antwerpen M. Grass G. (2020). Evaluation of a highly efficient DNA extraction method for *Bacillus anthracis* endospores. *Microorganisms* 8:763. 10.3390/microorganisms8050763 32443768 PMC7285266

[B15] Kubota H. Tsuji H. Matsuda K. Kurakawa T. Asahara T. Nomoto K. (2010). Detection of human intestinal catalase-negative, Gram-positive cocci by rRNA-targeted reverse transcription-PCR. *Appl. Environ. Microbiol.* 76 5440–5451. 10.1128/AEM.03132-09 20581195 PMC2918946

[B16] Kugeler K. J. Pappert R. Zhou Y. Petersen J. M. (2006). Real-time PCR for *Francisella tularensis* Types A and B. *Emerg. Infect. Dis.* 12 1799–1801. 10.3201/eid1211.060629 17283646 PMC3372352

[B17] Matsuda K. Tsuji H. Asahara T. Kado Y. Nomoto K. (2007). Sensitive quantitative detection of commensal bacteria by rRNA-targeted reverse transcription-PCR. *Appl. Environ. Microbiol.* 73 32–39. 10.1128/AEM.01224-06 17071791 PMC1797142

[B18] Maurin M. Castan B. Roch N. Gestin B. Pelloux I. Mailles A. (2010). Real-time PCR for diagnosis of oculoglandular tularemia. *Emerg. Infect. Dis.* 16 152–153. 10.3201/eid1601.090793 20031067 PMC2874363

[B19] Maurin M. Gyuranecz M. (2016). Tularaemia: clinical aspects in Europe. *Lancet Infect. Dis.* 16 113–124. 10.1016/S1473-3099(15)00355-2 26738841

[B20] Maurin M. Pelloux I. Brion J. P. Del Banõ J.-N. Picard A. (2011). Human tularemia in France, 2006-2010. *Clin. Infect. Dis. Off. Publ. Infect. Dis. Soc. Am.* 53 e133–e141. 10.1093/cid/cir612 22002987

[B21] Müller K. Daßen S. Holowachuk S. Zwirglmaier K. Stehr J. Buersgens F. (2021). Pulse-controlled amplification–a new powerful tool for on-site diagnostics under resource limited conditions. *PLoS Negl. Trop. Dis.* 15:e0009114. 10.1371/journal.pntd.0009114 33513140 PMC7875409

[B22] Pechous R. D. Sivaraman V. Stasulli N. M. Goldman W. E. (2016). Pneumonic plague: the darker side of *Yersinia pestis*. *Trends Microbiol.* 24 190–197. 10.1016/j.tim.2015.11.008 26698952

[B23] Rajanna C. Revazishvili T. Rashid M. H. Chubinidze S. Bakanidze L. Tsanava S. (2010). Characterization of pPCP1 plasmids in *Yersinia pestis* strains isolated from the former soviet union. *Int. J. Microbiol.* 2010:760819. 10.1155/2010/760819 21197443 PMC3010648

[B24] Rajerison M. Melocco M. Andrianaivoarimanana V. Rahajandraibe S. Rakotoarimanana F. Spiegel A. (2020). Performance of plague rapid diagnostic test compared to bacteriology: a retrospective analysis of the data collected in Madagascar. *BMC Infect. Dis.* 20:90. 10.1186/s12879-020-4812-7 32000692 PMC6993518

[B25] Randremanana R. Andrianaivoarimanana V. Nikolay B. Ramasindrazana B. Paireau J. Ten Bosch Q. A. (2019). Epidemiological characteristics of an urban plague epidemic in Madagascar, August-November, 2017: an outbreak report. *Lancet Infect. Dis.* 19 537–545. 10.1016/s1473-3099(18)30730-8 30930106 PMC6483974

[B26] Riehm J. M. Rahalison L. Scholz H. C. Thoma B. Pfeffer M. Razanakoto L. M. (2011). Detection of *Yersinia pestis* using real-time PCR in patients with suspected bubonic plague. *Mol. Cell. Probes* 25 8–12. 10.1016/j.mcp.2010.09.002 20933595

[B27] Tomaso H. Reisinger E. C. Al Dahouk S. Frangoulidis D. Rakin A. Landt O. (2003). Rapid detection of *Yersinia pestis* with multiplex real-time PCR assays using fluorescent hybridisation probes. *FEMS Immunol. Med. Microbiol.* 38 117–126. 10.1016/S0928-8244(03)00184-6 13129646

[B28] Uhlig S. Gowik P. (2018). Efficient estimation of the limit of detection and the relative limit of detection along with their reproducibility in the validation of qualitative microbiological methods by means of generalized linear mixed models. *J. Consum. Prot. Food Saf.* 13 79–87. 10.1007/s00003-017-1130-0

[B29] Vallès X. Stenseth N. C. Demeure C. Horby P. Mead P. S. Cabanillas O. (2020). Human plague: an old scourge that needs new answers. *PLoS Negl. Trop. Dis.* 14:e0008251. 32853251 10.1371/journal.pntd.0008251 PMC7451524

[B30] Wittwer C. T. Kusukawa N. (2004). “Real-time PCR,” in *Molecular Microbiology: Diagnostic Principles and Practice*, eds Persing D. H. Tenover F. C. Versalovic J. Tang Y. W. Unger E. R. Relman D. A. (Washington, DC: ASM Press), 71–84.

[B31] Woese C. R. (1987). Bacterial evolution. *Microbiol. Rev.* 51 221–271. 2439888 10.1128/mr.51.2.221-271.1987 PMC373105

[B32] Ziegler I. Vollmar P. Knüpfer M. Braun P. Stoecker K. (2021). Reevaluating limits of detection of 12 lateral flow immunoassays for the detection of *Yersinia pestis*, *Francisella tularensis*, and *Bacillus anthracis* spores using viable risk group-3 strains. *J. Appl. Microbiol.* 130 1173–1180. 10.1111/jam.14863 32970936

