# Supplementary material for: Development and Validation of Ribosomal RNA-Targeted Reverse Transcription Real-Time PCR Assays for the Sensitive and Rapid Diagnostics of High Consequence Pathogens
Source: Front Microbiol. 2021 Oct 29;12:738868. doi: 10.3389/fmicb.2021.738868 (PMC8589101; doi:10.3389/fmicb.2021.738868)
Supplement: Supplementary file 1 [file Data_Sheet_1.pdf]

Supplemental Material

Tab. S1 No cross-reactivity was observed with any of the tested bacteria, confirming specificity of the newly designed assays (ATCC: American Type Culture Collection; DSMZ: German Collection of Microorganisms and Cultures; NCTC: National Collection of Type cultures)

**Species**	**Strain**
Acinetobacter baumanii	DSMZ 7324
Bacillus anthracis	Vollum
Bacillus cereus	ATCC 10987
Bacillus globigii	DSMZ 7264
Bacillus thuringensis	DSMZ 2046
Brucella abortus	ATCC 23448
Burkholderia cepacia	NCTC 10744
Burkholderia mallei	Zagreb
Burkholderia pseudomallei	ATCC 23343
Burkholderia thailandensis	DSMZ 13276
Campylobacter jejuni	ATCC 29482
Candida albicans	ATCC 36232
Chlamydophila pneumoniae	ATCC 53592
Citrobacter freundii	DSMZ 30039
Clostridium perfringens	ATCC 12915
Clostridium sporogenes	DSMZ 795
Coxiella burnetii	Nine Mile
Eikenella corrodens	DSMZ 8340
Enterobacter aerogens	DSMZ 30053
Enterobacter faecalis	DSMZ 2570
Escherichia coli	ATCC 11303

**Species**	**Strain**
Haemophilus influenzae	ATCC 10211
Klebsiella pneumoniae	ATCC 13883
Legionella pneumophila	NCTC 10332
Listeria monocytogenes	DSMZ 12464
Moraxella catarrhalis	DSMZ 9143
Mycobacterium tubercolosis	
Neisseria meningitidis	Patient isolate
Proprionibacterium acnes	DSMZ 1897
Proteus mirabilis	DSMZ 788
Pseudomonas aeruginosa	ATCC 10145
Salmonella enterica typhii	20-3267
Serratia marcescens	DSMZ 1636
Shigella dysinteriae	B476
Staphylococcus aureus	DSMZ 19041
Staphylococcus epidermidis	DSMZ 1798
Stenotrophomoas maltophilia	ATCC 5131
Streptococcus pneumoniae	DSMZ 20566
Streptococcus pyogenes	DSMZ 20565
Vibrio cholerae	ATCC 15748
Yersinia enterocolitica	DSMZ 13030
Yersinia frederiksenii	ATCC 33641
Yersinia pseudotubercolosis	ATCC 29833

Tab. S2 Overview of various *F. tularensis* and *Y. pestis* strains, which were tested for DNA detection capabilities of the newly designed assay via rtPCR. (ATCC: American Type Culture Collection; DSMZ: German Collection of Microorganisms and Cultures; IMB: Bacterial strain collection of the Bundeswehr Institute of Microbiology; NCTC: National Collection of Type cultures)

**Species**	**Strain**
Francisella tularensis hispaniensis	DSMZ 22475
Francisella tularensis holartica japonica	IMB FO700285
Francisella noatunesis noatunesis	DSMZ 23596
Francisella philomiragia	ATCC 25016
Francisella tularensis mediasiatica	FSC 149
Francisella tularensis novicida	FSC 595
Francisella tularensis novicida	FSC 156
Yersinia pestis antiqua 1.ANT	IMB L3-2996
Yersinia pestis antiqua 2. ANT	IMB L3-3004
Yersinia pestis medievalis	IMB L3-2536
Yersinia pestis orientalis	IMB L3-2540
Yersinia pestis orientalis	IMB L3-2992
Yersinia pestis pestoides	IMB L3-2997

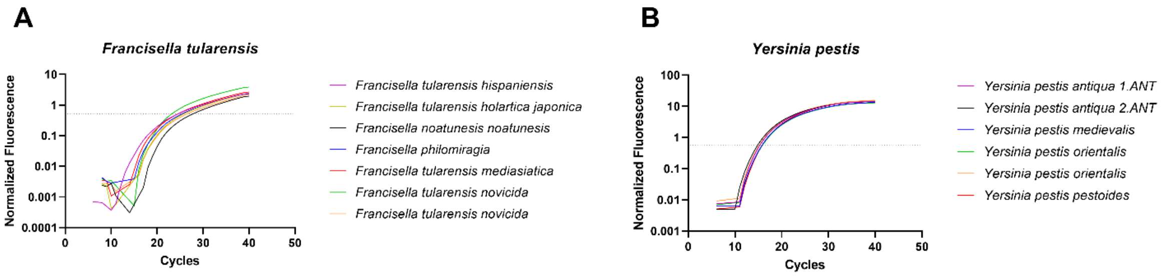

Figure S1 Real time PCR (rtPCR) detection of several (A) *Francisella tularensis* (*F. tularensis)* and (B) *Yersinia pestis* (*Y. pestis)* strains by using the newly designed assay, confirming the ability to detect in general *F.tularensis* and *Y.pestis* DNA.

The rtPCR was conducted as described in the part for Materials and Methods. 5 µl DNA was added to each rtPCR. For *Y. pestis* rtPCR, the competitive probe was included (*Y. pestis* probe:competitive probe 1:3). The first five cycles were excluded and the software calculated the threshold automatically. All measurements were performed in duplicates and repeated three times. The mean was estimated for each strain and is monitored for (A) *F. tularensis* and (B) *Y.pestis* strains.
